# Leprosy in the 21^st^ century: a comprehensive review of immunological mechanisms, diagnosis, and treatment

**DOI:** 10.1590/S1678-9946202567074

**Published:** 2025-11-03

**Authors:** Luis Alberto Ribeiro Froes, Mirian Nacagami Sotto, Maria Angela Bianconcini Trindade

**Affiliations:** 1 Universidade de São Paulo, Faculdade de Medicina, Departamento de Patologia, São Paulo, São Paulo, Brazil; 2 Universidade de São Paulo, Faculdade de Medicina, Departamento de Dermatologia, São Paulo, São Paulo, Brazil; 3 Universidade de São Paulo, Faculdade de Medicina, Hospital das Clínicas, Laboratório de Investigação em Dermatologia e Imunodeficiências (LIM-56), São Paulo, São Paulo, Brazil; 4 Secretaria de Estado da Saúde, Instituto de Saúde, São Paulo, São Paulo, Brazil

**Keywords:** Leprosy, Mycobacterium leprae, Immunopathology, Neuropathy, Multidrug therapy

## Abstract

Leprosy remains a significant public health challenge, with approximately 182,815 new cases annually. This review synthesizes current knowledge on pathogenesis, diagnosis, and treatment, emphasizing recent advances. We discuss the immunological spectrum, ranging from Th1-dominant tuberculoid forms to Th2/regulatory-dominant lepromatous forms, and highlight the roles of B-lymphocyte, inflammasome activation, and Schwann cell antigen presentation in granuloma formation and neural damage. Leprosy reactions—type 1 and 2—arise from distinct immunopathological mechanisms triggered by various factors, with emerging evidence pointing to the involvement of Th17 and regulatory B cells. Diagnostic advances include molecular techniques, nerve ultrasonography, monofilament testing, and serological assays that complement traditional approaches. Treatment is based on multidrug therapy, with country-specific adaptations such as Brazil’s PQT-U, although challenges with adherence and resistance persist. Alternative regimens may include minocycline, clarithromycin, and fluoroquinolones, with bedaquiline showing promising results. Prophylactic strategies include BCG vaccination and the debated use of single-dose rifampicin for contacts. Management of reactional states employs corticosteroids, thalidomide, with biologics and JAK inhibitors reserved for refractory cases. Genetic and epigenetic factors, including TLR polymorphisms and HLA variants, influence susceptibility and outcomes. Despite recent progress, delayed detection, stigma, and inadequate follow-up remain barriers. Comprehensive approaches integrating early diagnosis, active case finding, chemoprophylaxis, stigma reduction, and rehabilitation are essential. The elimination of leprosy requires sustained commitment, improved resource access, and ongoing research into host-pathogen interactions. This review offers clinicians and researchers an updated understanding to support global control strategies.

## INTRODUCTION

Leprosy remains a significant public health concern, with approximately 182,815 new cases reported in 2023^1^. Despite its ancient origins, the disease continues to affect socioeconomically vulnerable regions, where inadequate housing conditions and limited access to healthcare hinder effective control of *Mycobacterium leprae* and, to a lesser extent, *Mycobacterium lepromatosis* ^2^. While transmission occurs primarily via the upper respiratory tract, evidence supports alternative routes, including blood-feeding insects and tattooing^3,4^.

The World Health Organization (WHO) reported that 5.7% of new cases in 2023 were diagnosed in children, indicating ongoing transmission. This figure, along with the long incubation period of the disease, underscores the need for continuous surveillance^1^. In several regions considered to have low endemicity, studies have revealed significant underreporting of cases, highlighting the importance of active case finding and contact tracing^5^. Despite the availability of free multidrug therapy, challenges persist, especially delayed diagnosis in advanced cases, as well as stigmatization and neurological impairment. Consequently, epidemiological surveillance is essential for early detection and implementation of targeted interventions in high-risk communities to limit bacillary spread, interrupt transmission, prevent disabilities, and improve treatment adherence^6^.

Immunologically, leprosy manifests along a spectrum. The tuberculoid pole (TT) is characterized by strong cellular immune responses and low bacillary loads, while the lepromatous pole (LL) exhibits predominantly humoral immune responses with high bacterial burdens^7^. Between these extremes lie borderline forms (BT, BB, and BL), presenting intermediate immunological, histological, and clinical features. In multibacillary forms (BB to LL), the high bacillary burden stimulates robust antibody production; these antibodies, however, fail to halt bacterial proliferation^7^. Nerve damage occurs due to the pathogen’s affinity for Schwann cells, leading to inflammatory and degenerative changes in the peripheral nervous system that increase the risk of deformities^8^.

Progress in leprosy control has been achieved with multidrug therapy, active surveillance, health education, and contact tracing. However, sustained reductions in incidence rates require broader social awareness and coordinated efforts to address stigma. Early diagnosis is crucial, as timely treatment during initial stages reduces long-term sequelae and prevents permanent disabilities^6,9^. Recent initiatives have expanded post-exposure prophylaxis strategies, including BCG vaccination and, in some regions, single-dose rifampicin for contacts of multibacillary cases, although the efficacy of the latter remains under debate^10,11^.

This review provides an updated synthesis of clinical, immunological, and epidemiological aspects of leprosy, highlighting recent advances in prophylactic and therapeutic strategies. Additionally, we examine persistent challenges hindering sustained reductions in disease incidence, emphasizing the importance of early diagnosis, contact tracing, and public health interventions for effective disease control.

### Etiology and pathogenesis

#### The etiological agent

Leprosy is a mycobacterial infection characterized by high infectivity and low pathogenicity, with *Mycobacterium leprae* as the primary causative agent. To a lesser extent, *Mycobacterium lepromatosis* is also recognized as a causative organism. Initially identified in Mexico in 2008, *M. lepromatosi*s has been associated with both multibacillary and paucibacillary forms of the disease^2^. *M. leprae* exhibits tropism for cutaneous macrophages and Schwann cells in the peripheral nerves. Studies have shown that, in addition to humans, armadillos (*Dasypus novemcinctus*) can become naturally infected and act as reservoirs, thereby expanding the range of potential hosts and transmission routes^12,13^.

The cell wall of *M. leprae* consists of a lipid-rich matrix, predominantly mycolic acids, in which phenolic glycolipid I (PGL-I) emerges as a critical virulence factor. PGL-I promotes bacillary uptake by macrophages via interaction with CR1, CR3, and CR4 receptors and also binds to specific targets such as α-dystroglycan on Schwann cells, contributing to myelin sheath damage and the development of nerve lesions^7,14^.

Transmission occurs primarily via the respiratory route, with the upper airways as the main entry point^3^. Nevertheless, other routes have been proposed, including transmission by blood-feeding insects and inoculation during tattooing procedures, although these are less well-documented^4^. The extended incubation period—ranging from months to decades—further complicates the establishment of clear epidemiological links.

#### Innate immune response

The innate immune response is the first line of defense against *M. leprae* infection and involves a range of cellular and molecular mechanisms, especially pattern recognition receptors (PRRs) such as Toll-like receptors (TLRs). Among these, TLR2 and TLR4 are crucial in detecting microbial components and initiating signaling pathways that drive the production of pro-inflammatory cytokines. Studies indicate that mutations or polymorphisms in the TLR2 gene may increase susceptibility to the lepromatous form of the disease, which is associated with higher bacillary loads^15,16^.

Macrophages and other antigen-presenting cells phagocytose the bacillus, a process enhanced by opsonization with complement component C3 and CR1, CR3, and CR4 receptors. Once internalized, *M. leprae* persists within the intracellular compartment, partly due to its extremely slow replication rate (approximately every 14 days) and its ability to modulate host cell pathways that inhibit lysosome formation and fusion. This favors the formation of foamy macrophages (or foam cells), which accumulate lipids and exhibit reduced microbicidal activity, including diminished production of nitric oxide (NO) and reactive oxygen species (ROS)^17^.

This functional impairment aligns with the polarization of macrophages into two distinct subsets: M1 and M2. In tuberculoid forms, macrophages typically exhibit an M1 phenotype, characterized by enhanced microbicidal capacity, inducible nitric oxide synthase (iNOS) expression, and the production of pro-inflammatory cytokines such as TNF-α and IL-12. Conversely, in lepromatous forms, macrophages predominantly display an M2 phenotype, associated with increased secretion of IL-10 and TGF-β, ineffective bacillary killing, and maintenance of a permissive environment for *M. leprae* proliferation^7^.

The inflammasome, a cytosolic protein complex responsible for caspase-1 activation and subsequent release of IL-1β and IL-18, also contributes to the innate immune response against mycobacteria. However, in leprosy, reports indicate that even when the inflammasome is activated in lepromatous lesions, its activity is insufficient to eliminate the pathogen, possibly due to bacillary evasion mechanisms or excessive immunoregulatory suppression that dampens inflammation^18^.

Schwann cells are also critical in the pathogenesis of leprosy. Beyond being the primary target of *M. leprae* in the peripheral nervous system, they can present bacillary antigens to CD4+ T cells, thereby triggering a localized inflammatory response. However, this process also contributes to tissue damage and demyelination, making Schwann cells central in leprosy-associated neuropathy^19^.

#### Adaptive immune response

The adaptive immune response, primarily mediated by T and B lymphocytes, is a major determinant of the clinical spectrum of leprosy, which ranges from tuberculoid (TT) to lepromatous (LL) forms. TT is associated with a robust cell-mediated immune response and low bacillary load, while LL is dominated by a humoral response and high bacillary burden.

In TT, there is a predominance of Th1-type cytokines such as IFN-γ, IL-2, and TNF-α, which activate macrophages and promote the formation of compact granulomas capable of restricting *M. leprae* proliferation. These granulomas—composed primarily of activated macrophages, epithelioid cells, and T lymphocytes—function as effective barriers against bacillary dissemination^20^. Within this context, increased inducible nitric oxide synthase (iNOS) expression further enhances macrophage microbicidal capacity^7^.

Conversely, in LL, Th2-type cytokines such as IL-4, IL-5, and IL-10 predominate. This cytokine profile inhibits effective macrophage activation and reduces the production of microbicidal mediators, facilitating widespread bacillary dissemination. Patients in this group typically exhibit high titers of circulating antibodies against *M. leprae* antigens; however, these antibodies are largely ineffective in controlling infection^7^.

Despite the intensity of the humoral response, *M. leprae* persists within macrophages and Schwann cells. The elevated expression of IL-10 and TGF-β in LL lesions establishes an immunosuppressive microenvironment, inhibiting effective cellular responses^7^.

Beyond the classical Th1/Th2 dichotomy, other CD4+ T cell subsets are relevant in leprosy immunopathogenesis, particularly Th17 cells. These cells produce IL-17, IL-21, and IL-22, mediating neutrophil recruitment and sustaining chronic inflammatory responses. Higher IL-17 levels are generally observed in tuberculoid lesions and are associated with stronger cellular responses^21^.

Conversely, regulatory T cells (Tregs) are known to modulate immune responses by secreting suppressive cytokines such as IL-10 and TGF-β. In multibacillary forms, expansion of Treg populations is frequently observed and is thought to impair cell-mediated immunity, thereby facilitating bacillary proliferation^22^.

B cells also contribute to leprosy pathogenesis. In the lepromatous form, abundant plasma cells produce antibodies, which, despite being plentiful, lack strong microbicidal activity. In contrast, in paucibacillary forms, B lymphocytes are present in granulomatous areas and may contribute to local immune coordination, although their precise role remains unclear^23^.

A recent study examined the distribution of B cell subpopulations across different clinical and reactional forms of leprosy, revealing that both their abundance and functional profiles vary with disease presentation. In TT lesions, CD20+ B cells were predominantly localized around granulomas, with B-1 and Be1 subsets predominating. These subsets are associated with innate immune responses and Th1 polarization, and were also prominent in Type 1 Reactions (T1R), suggesting a role in amplifying cell-mediated responses. Marginal zone B cells (MZ B), which participate in rapid responses against T-independent antigens, were likewise found in T1R lesions, potentially supporting early local immune activation. Although relatively scarce, regulatory B cells (Bregs) were more frequent in T1R, suggesting a subtle role in moderating inflammation. These findings indicate that B cells and their subsets are more significant in leprosy than previously recognized, particularly in forms associated with Th1-type responses, notably in T1R^24^.

#### Immunology of leprosy reactions

Leprosy reactions are acute inflammatory episodes that may occur at any point during the disease and are categorized into Type 1 Reaction (T1R) and Type 2 Reaction (T2R).

T1R is characterized by an exacerbation of cell-mediated immunity and is most commonly associated with borderline forms (BT, BB, and BL). It involves increased production of Th1 cytokines such as IFN-γ, TNF-α, and IL-2, leading to intense inflammation with infiltration of macrophages and CD4+ T lymphocytes. Clinically, T1R manifests as an acute exacerbation of pre-existing skin lesions, since the inflammatory response impacts previously affected areas rather healthy skin. Patients frequently experience painful neuritis, which, even with prompt corticosteroid treatment to mitigate tissue damage, can result in severe and irreversible nerve impairment^19^. The inflammatory infiltrate is characterized by M1-type epithelioid macrophages, which contribute to the intense cellular immune response and enhanced microbicidal activity typical of T1R^7^.

In contrast, T2R predominantly affects patients with multibacillary forms (BB, BL, and LL) and is marked by the sudden appearance of painful erythematous nodules, often accompanied by systemic symptoms such as fever and malaise. Although the exact mechanism is still debated—whether mediated by immune complexes (type III hypersensitivity) or by excessive cytokine release—T2R is associated with elevated levels of TNF-α, Th2 cytokines, and circulating IgG and IgM. Deposition of immune complexes in blood vessels may result in vasculitis and tissue damage, specifically affecting vessels previously parasitized by *M. leprae*. Notably, T2R selectively affects previously infected tissues while sparing non-parasitized areas^7^.

Management of leprosy reactions primarily involves corticosteroids such as prednisone to suppress inflammation. In severe or recurrent cases of T2R, thalidomide may be employed due to its ability to inhibit TNF-α and other pro-inflammatory cytokines. Anti-TNF agents, such as infliximab and etanercept, have also been reported in refractory T2R cases, although this approach remains limited in clinical practice^25^.

#### Immunopathogenesis of nerve damage

Peripheral nerve damage in leprosy results from a combination of direct *Mycobacterium leprae* invasion, exaggerated inflammatory responses, and degenerative processes within neural tissue^14^. Inflammatory infiltrates, primarily composed of macrophages and lymphocytes, contribute to neural damage by the release of cytotoxic mediators and tissue-destructive enzymes^8^. Additionally, intraneural inflammatory events during leprosy reactions (T1R and T2R) can cause partial or complete destruction of neural tissues, significantly contributing to neuropathic complications of the disease^8,19^.

The entry of *M. leprae* into Schwann cells via PGL-1^14^ triggers a cascade of neural damage, including demyelination, activation of matrix metalloproteinases, and release of inflammatory mediators. This cascade leads to both axonal degeneration and perpetuation of local inflammation, ultimately causing the characteristic neuropathy of leprosy^14^.

Simultaneously, inflammatory mediators such as IL-1β and TNF-α—released by T lymphocytes and macrophages—stimulate NADPH oxidase activity, increasing ROS production. This oxidative burst induces mitochondrial dysfunction in Schwann cells, contributing to axonal degeneration. Moreover, the hypoxic microenvironment generated by excessive oxidative activity impairs regenerative processes and disrupts nerve impulse transmission^8^.

Clinically, these pathophysiological processes manifest as sensory and motor deficits, leading to deformities and disability. In TT, the strong inflammatory response may result in granuloma formation that compresses nerve fibers, whereas in LL, diffuse bacillary infiltration and ineffective immune responses promote progressive neural function loss. Thus, the accumulation of neurotoxic mediators, combined with matrix metalloproteinase (MMP) activity and oxidative stress, are relevant in peripheral nerve degeneration^26^.

#### Genetic and epigenetic factors

The high proportion of pseudogenes (~50% of the genome) renders *M. leprae* heavily dependent on host metabolism. These findings suggest that clinical diversity in leprosy largely stems from interindividual variation in host immune responses, shaped by a complex interplay of genetic and epigenetic factors. Genome-wide association studies (GWAS) have identified genes such as PARK2, LRRK2, MRC1, and VDR as key modulators of immune response^27^. Additionally, polymorphisms within the HLA complex and epigenetic modifications—such as differential DNA methylation and microRNA activity—significantly contribute to disease variability^28-30^.

The interplay between these genetic variants and epigenetic factors shapes the magnitude and profile of the host’s immune response, ultimately influencing clinical progression. Understanding these mechanisms is essential for explaining the diversity of leprosy manifestations and identifying biomarkers to assess disease risk and predict clinical outcomes.

## Clinical manifestations and diagnostic approaches

### Clinical manifestations

The Madrid classification system categorizes leprosy into four main clinical forms: indeterminate, tuberculoid, borderline, and lepromatous. This classification integrates clinical, histopathological, and bacteriological criteria, providing a practical framework that correlates host immunological status with disease presentation. However, at the operational level, many countries adopt a simplified classification based on the number of skin lesions, nerve involvement, and slit-skin smear results—paucibacillary (PB) and multibacillary (MB)—to guide therapeutic decisions and facilitate epidemiological surveillance^31^.

### Clinical forms and operational classification

**Figure 1:**
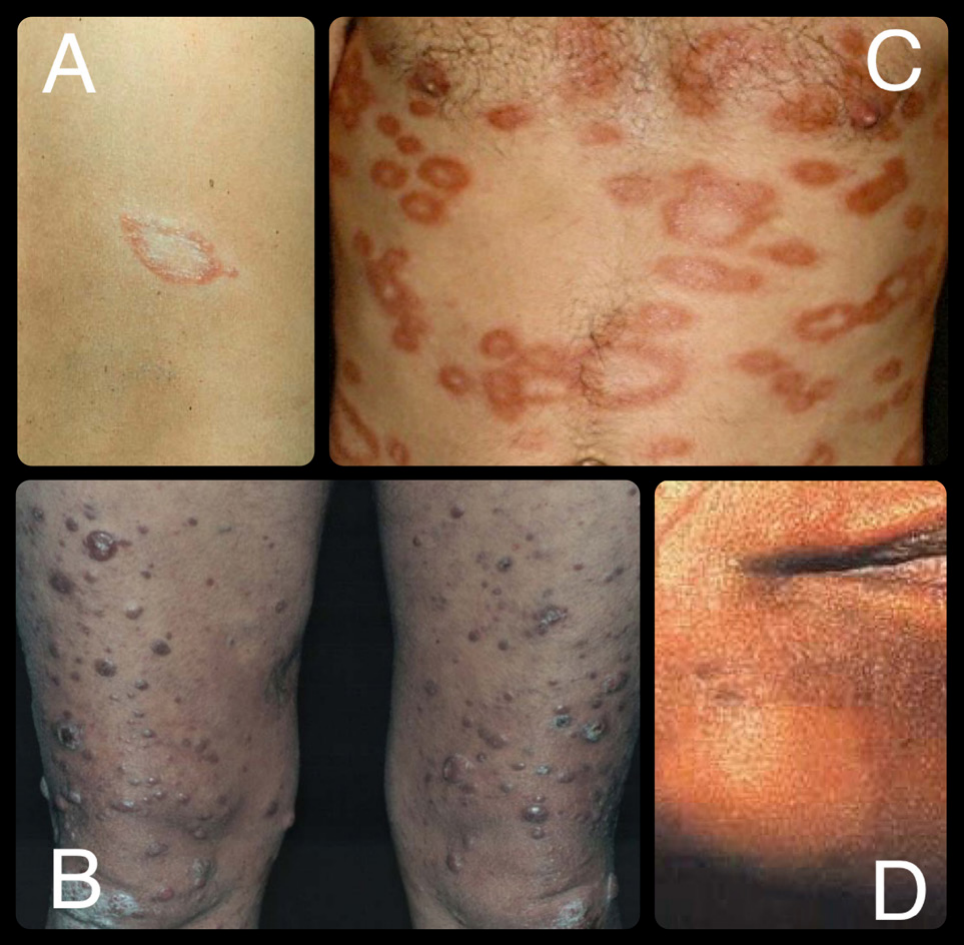
Clinical forms of leprosy: (A) Tuberculoid leprosy: a well-demarcated hypopigmented lesion with reduced sensitivity; (B) Lepromatous leprosy: multiple diffuse papulonodular lesions with high bacillary load; (C) Borderline leprosy: annular plaques with raised borders and central clearing; (D) Indeterminate leprosy: ill-defined hypopigmented macule with subtle sensory alteration.

In the TT form (Figure 1A), effective immunological control limits *M. leprae* multiplication, resulting in a small number of well-demarcated lesions, often accompanied by hypoesthesia, alopecia, xerosis, and by a typically negative bacteriological index. In contrast, the LL form reflects a failure of cellular immunity, leading to diffuse skin infiltration and high bacteriological indices (Figure 1B), while the BB form presents intermediate characteristics between the two poles (Figure 1C)^32^.

For treatment purposes, the WHO recommends an operational classification into paucibacillary (PB) and multibacillary (MB) forms. PB cases are defined by up to five skin lesions, a negative slit-skin smear, and involvement of only one nerve trunk. MB cases encompass patients with six or more lesions, a positive bacilloscopy, or involvement of two or more nerve trunks. This classification guides treatment duration in countries that have not adopted the uniform six-month multidrug therapy regimen, such as Brazil^33^.

Indeterminate leprosy represents an early stage in which the host immune response is not yet fully established. Lesions are subtle, with ill-defined borders and mild sensory changes (Figure 1D)^32^. In most cases, this form progresses to one of the aforementioned categories depending on whether a Th1 or Th2 response predominates, although spontaneous resolution may also occur in some instances^34^.

### Leprosy reactions

Leprosy reactions are acute, unpredictable inflammatory events that may occur before, during, or after treatment, resulting from fluctuations in the host immune response to the bacillus. These reactions can affect any tissue previously infected by *M. leprae* ^35^.

**Figure 2:**
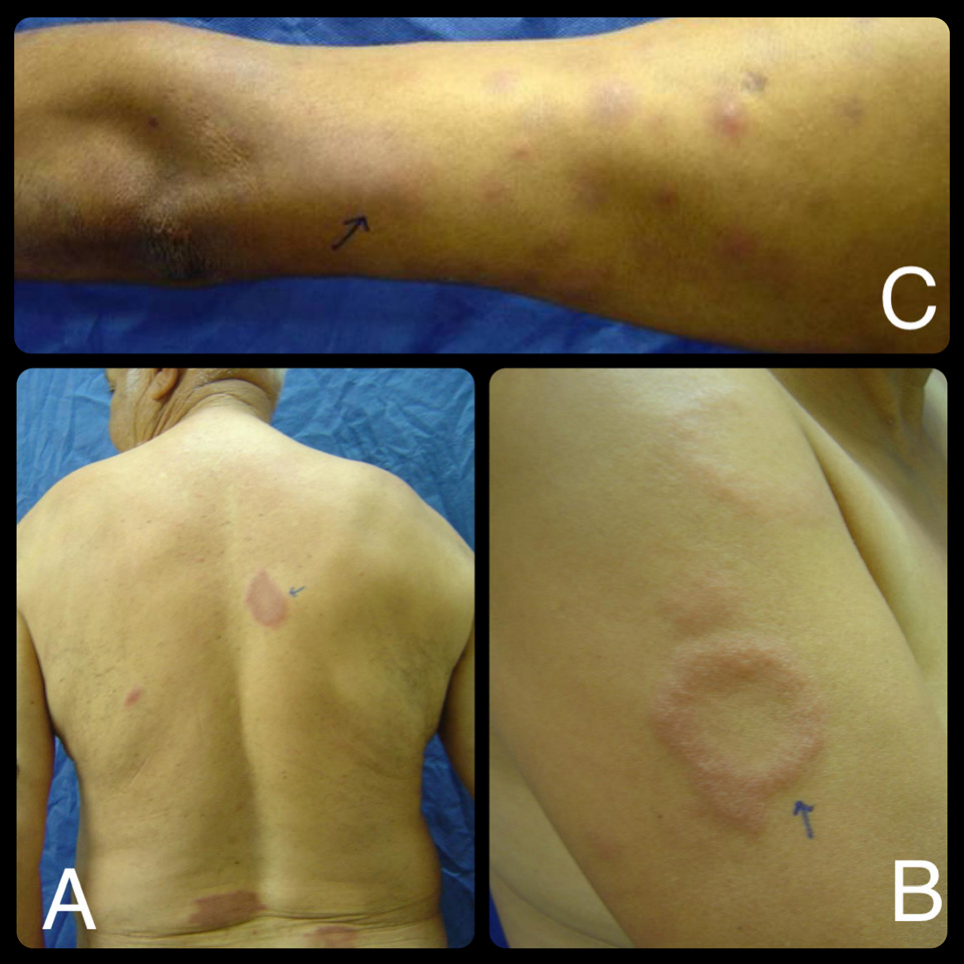
Leprosy reactions: (A and B) Type 1 reaction (T1R): reactivation of existing lesions with marked erythema and edema, accompanied by acute neuritis; (C) Type 2 reaction (T2R): painful subcutaneous nodules with systemic inflammation.

T1R typically occurs in borderline forms and is associated with increased cellular immunity against *M. leprae*. In the skin, this manifests as an exacerbation of pre-existing lesions and the emergence of new erythematous areas. Acute neuritis may also occur, characterized by intense pain and rapid deterioration of neural function. Nerve damage results from granulomatous infiltration around neural structures, interstitial edema, axonal compression, and segmental demyelination, which can progress to necrosis without immediate intervention (Figures 2A and 2B)^36^.

T2R is observed in multibacillary patients and manifests as persistent systemic inflammation mediated by immune complex deposition and neutrophil activation. Clinically, it presents painful subcutaneous nodules, fever, and multisystem involvement, including more diffuse neural impairment (Figure 2C). This process promotes prolonged neuritis and progressive nerve degeneration, significantly increasing the risk of functional disability if not promptly managed. T2R may also lead to dysfunction of both the peripheral nervous system and multiple organs, including the testes, eyes, kidneys, liver, spleen, bone marrow, and oropharynx^7^.

Leprosy reactions can arise at any disease stage, even after treatment completion, and may be triggered by stress, coinfections, and hormonal factors^37,38^. Early recognition and appropriate treatment of these reactions are essential to minimize long-term sequelae^19^. Hence, continuous neurological monitoring—assessing sensory function, muscle strength, and signs of neuritis—is critical to detect reactional changes and initiate timely therapy^32^.

### Microbiological diagnosis

Slit-skin smear microscopy remains the most widely used laboratory test in leprosy. Samples are collected from cooler body areas—such as earlobes, elbows, and suspicious lesions—in which bacilli are more likely to be found, and are stained using the modified Ziehl-Neelsen method to detect acid-fast bacilli. The bacteriological index (BI), ranging from 0 to 6+, reflects bacillary load and is useful for operational classification. While highly specific, this method has limited sensitivity in PB patients, who typically exhibit a low density of *M. leprae* ^39^.

To improve detection, especially in smear-negative cases, molecular techniques such as PCR and qPCR are employed to identify specific DNA fragments of the bacillus^40^. These techniques show good sensitivity for detecting *M. leprae* DNA even in paucibacillary samples. However, their routine implementation in endemic settings is restricted by logistical barriers, including dependence on imported reagents, advanced laboratory infrastructure, and trained personnel. These constraints restrict feasibility in public health services in low- and middle-income countries^41^. Nonetheless, population-based data from urban low-endemic regions suggest that such expanded tools could reveal a higher-than-expected disease burden, supporting the argument for broader implementation despite these barriers^42^.

In pure neural forms of leprosy, diagnosis is particularly challenging due to the absence of cutaneous lesions and negative slit-skin smears. However, a comprehensive approach combining neurophysiological, serological, and molecular techniques—such as electroneuromyography, anti-PGL-1 antibody detection, and qPCR for *M. leprae* DNA—has demonstrated high diagnostic accuracy in these cases^43^.

### Histopathological diagnosis

**Figure 3:**
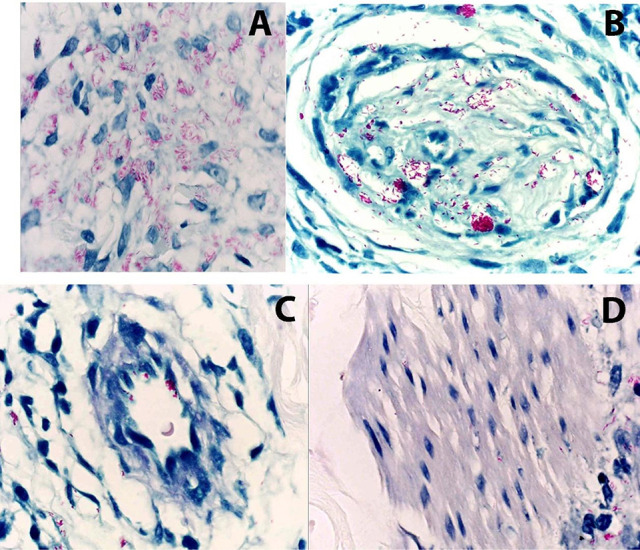
Fite-Faraco staining reveals *Mycobacterium leprae* bacilli within different host cells: macrophages (A), Schwann cells (B), endothelial cells (C), and arrector pili muscle (D).

Histopathological examination provides in-depth insights into the inflammatory response and bacillary distribution in tissue samples. The Fite-Faraco staining technique is employed to detect *M. leprae*, assessing bacillary load and the intensity of inflammatory infiltrates around skin appendages and nerve bundles (Figure 3).

**Figure 4:**
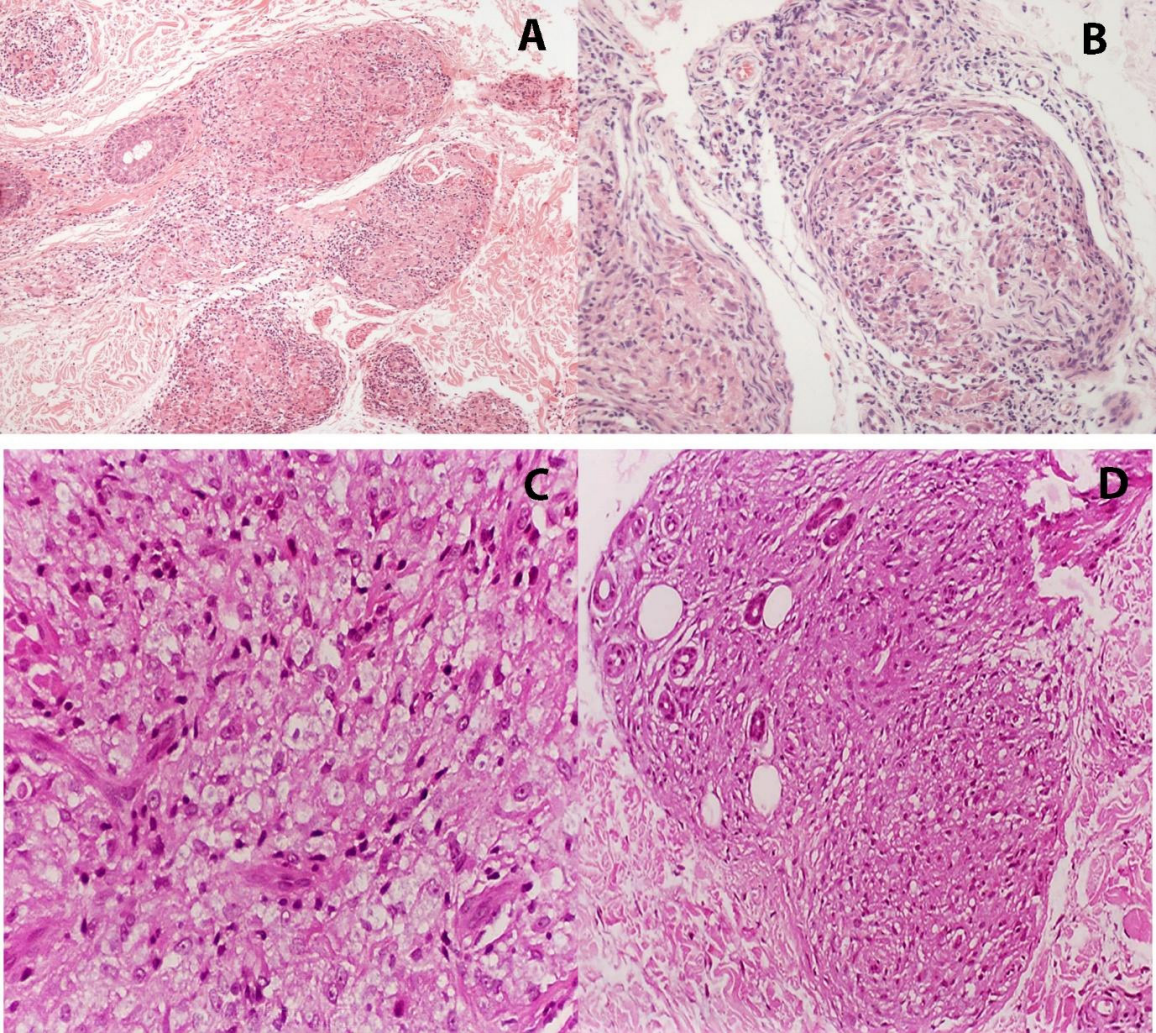
Tuberculoid leprosy showing well-formed epithelioid granulomas surrounding vasculoneural structures in the dermis (A), and granulomatous infiltration within a nerve bundle (B). In contrast, lepromatous leprosy is marked by dense infiltration of foamy macrophages (C), extending to adnexal structures such as sweat glands (D). Hematoxylin and eosin stain.

In tuberculoid leprosy, histological patterns are defined by well-formed granulomas—sometimes extending to the epidermis—composed predominantly of epithelioid cells, Langhans-type multinucleated giant cells (Figures 4A and 4B), and a dense peripheral rim of CD4+ T lymphocytes. The paucity of bacilli reinforces the effectiveness of the host immune response, and this histopathological profile closely correlates with clinical features such as sharply demarcated annular plaques, sensory loss, and, in some cases, associated alopecia and anhidrosis^32^.

Conversely, lepromatous leprosy reveals a diffuse infiltrate of foamy macrophages—known as Virchow cells—with abundant bacilli, globi formation, and poorly organized granulomas. Distinctive morphological features, such as the “grenz zone” (a subepidermal layer spared from infiltration), and, in some cases, onion-skin perineural lamination reflect the failure of cellular immunity and chronic nerve involvement (Figure 4C and 4D). Clinically, this form presents with multiple extensive lesions, including macules, papules, nodules, and plaques, often affecting cooler areas such as the face, ears, and extremities^44^.

A rare complication of untreated diffuse lepromatous leprosy is Lucio’s phenomenon, characterized by painful, irregularly shaped skin ulcers resulting from vascular thrombosis and cutaneous infarction. Histopathological findings include endothelial proliferation and thrombosis of medium-sized vessels, typically without a prominent neutrophilic infiltrate^7^.

**Figure 5:**
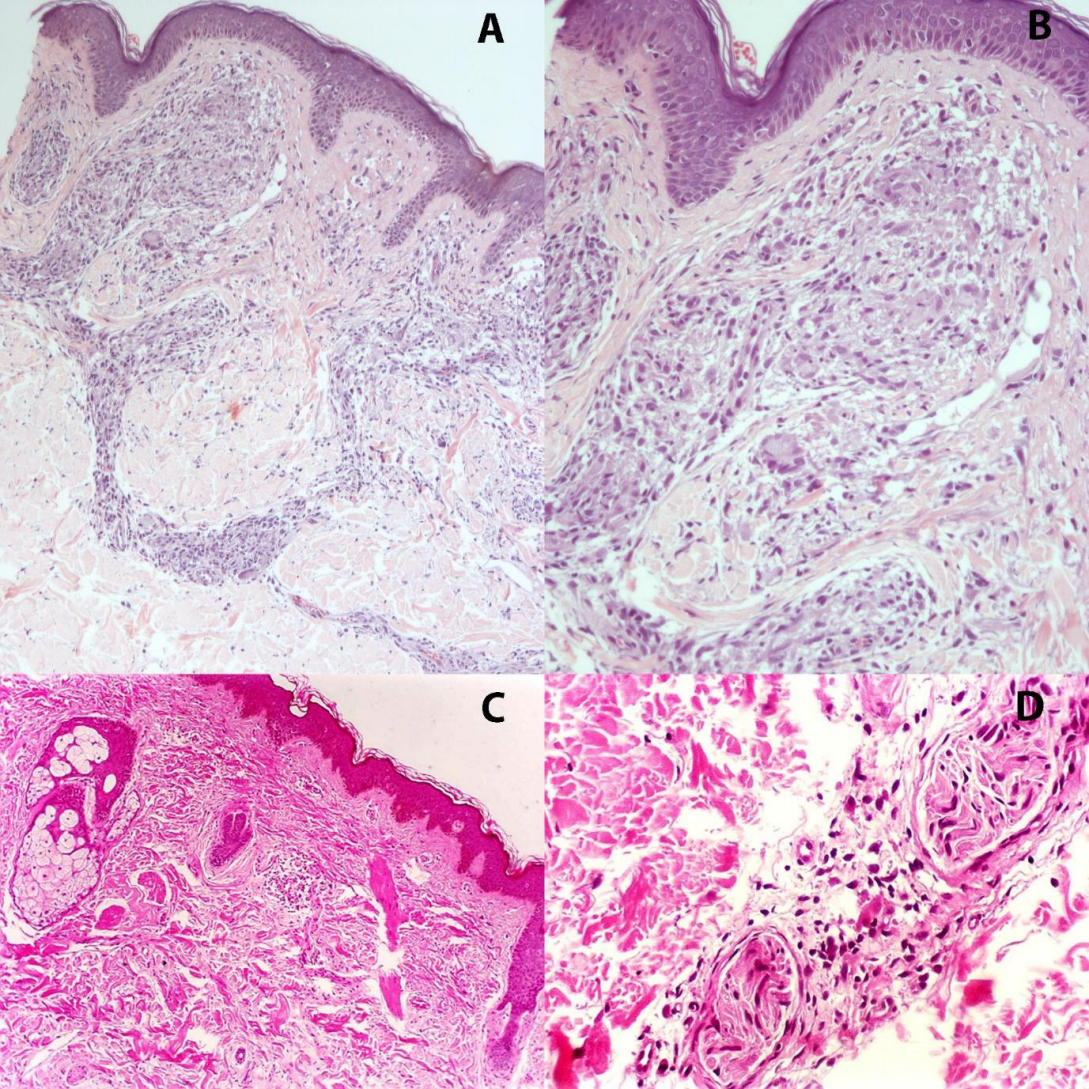
Borderline leprosy characterized by loosely organized granulomas (A), with mild lymphocytic infiltration and occasional multinucleated giant cells (B). Indeterminate leprosy shows a mild, nonspecific lymphohistiocytic infiltrate (C), sometimes associated with perineuritis (D). Hematoxylin and eosin stain.

Borderline forms display a mixed histological pattern, combining features of both tuberculoid and lepromatous poles (Figures 5A and 5B). In these cases, granulomas exhibit variable organization, and bacillary counts fluctuate depending on the position within the disease spectrum^17^.

In indeterminate leprosy, the inflammatory infiltrate is mild and predominantly lymphohistiocytic, typically surrounding skin appendages and nerve fibers, without well-formed granulomas or significant bacillary presence (Figures 5C and 5D) Clinically, this early-stage form manifests as hypopigmented macules with reduced sensation, decreased hair density, and diminished sweating. It may resolve spontaneously or progress to any of the classic forms, depending on the host’s immune response^17^.

The histoid variant, although less common, is a distinctive manifestation of lepromatous leprosy. It presents with papulonodular lesions composed of spindle-shaped macrophages and a high bacillary load, typically without globi formation. Historically associated with relapse following dapsone monotherapy, this variant is now observed in both relapse and *de novo* cases despite multidrug therapy, and displays unique clinical and histopathological features that assist in differentiating disease forms^45^.

Beyond disease classification, histopathological examination is an important tool to assess treatment response and detect disease recurrence. The presence of acid-fast bacilli and specific inflammatory patterns in tissue samples can help distinguish treatment failure, relapse, and residual post-treatment changes, as exemplified by the histoid variant, which may occur in both relapse and *de novo* cases^45^.

**Figure 6:**
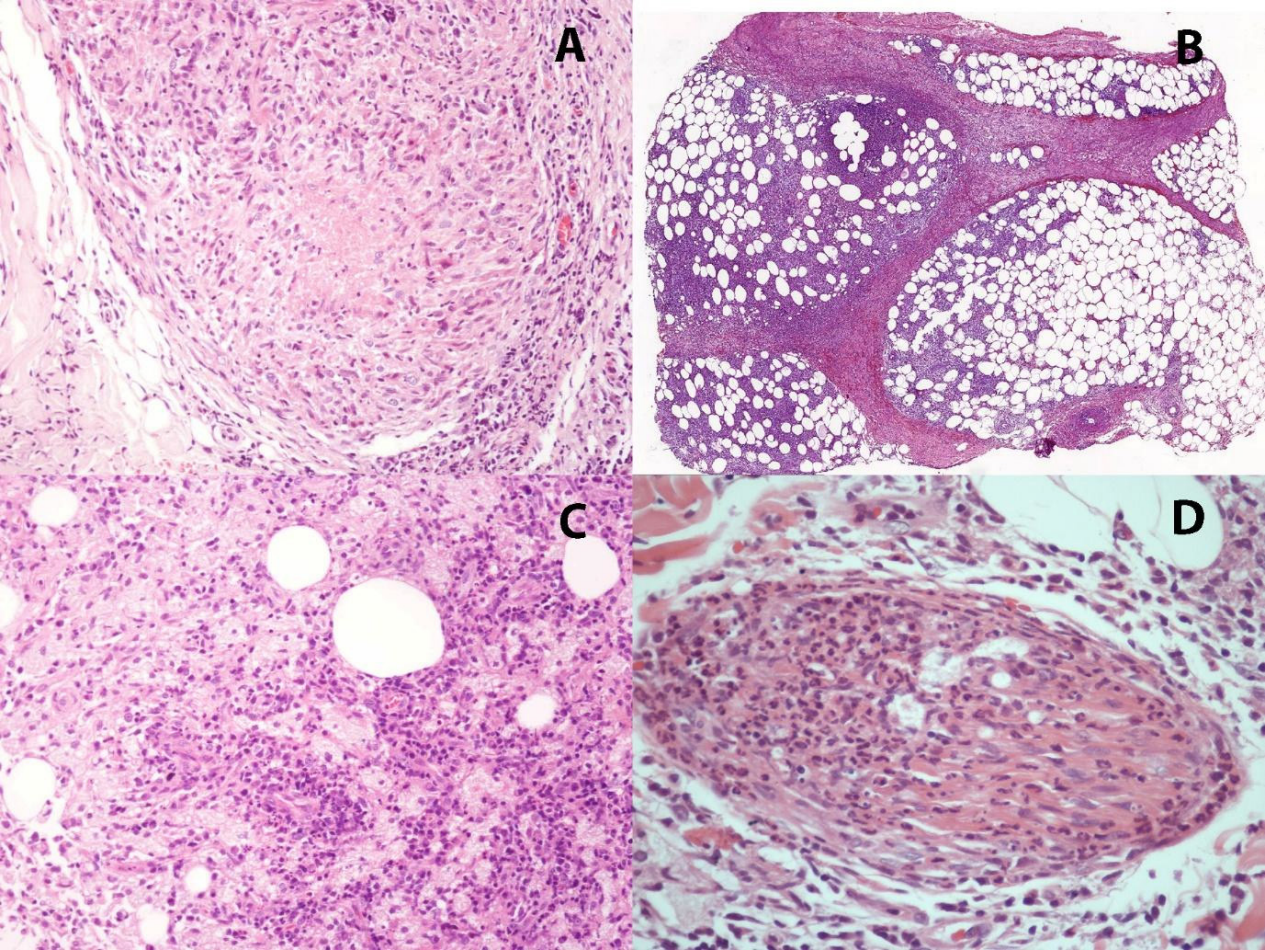
Type 1 reaction with granulomatous inflammation and focal necrosis (A). Type 2 reaction showing lobular panniculitis (B), with neutrophilic infiltrates interspersed among macrophages (C). Nerve involvement in type 2 reaction is illustrated by neutrophilic exudation within a nerve bundle (D). Hematoxylin and eosin stain.

In addition to classifying the clinical spectrum, histopathological evaluation is essential to identify leprosy reactions—acute exacerbations of the immune response. In T1R, granulomatous inflammation becomes more pronounced, with interstitial edema and occasionally focal necrosis, particularly around nerve bundles (Figure 6A). These changes can lead to rapid functional deterioration and require immediate medical intervention^18^.

In contrast, T2R is characterized by neutrophilic infiltration and immune complex deposition in affected tissues, manifesting as panniculitis in adipose tissue, vasculitis in blood vessels, or neuritis in nerves (Figures 6B and 6C), resulting in painful, widespread lesions that may ulcerate. In more severe cases, bullae and deeper ulcerations may develop, characterizing necrotizing erythema nodosum^7^. Systemic involvement can further exacerbate neural damage (Figure 6D), often requiring hospitalization^46^.

### Immunological diagnosis

Immunological diagnosis of leprosy primarily involves detecting antibodies against specific *Mycobacterium leprae* antigens, particularly phenolic glycolipid I (PGL-1)^47^. The presence of anti-PGL-1 antibodies indicates exposure to the bacillus but cannot distinguish active infection from prior contact. Since most individuals exposed to *M. leprae* do not develop leprosy, seropositivity alone is not diagnostic. Additionally, in PB patients, the predominant cell-mediated immune response suppresses antibody production, further reducing the sensitivity of serological tests^47^.

Conversely, MB patients typically exhibit higher anti-PGL-1 antibody titers due to impaired cellular immunity, which facilitates bacillary proliferation and greater immunoglobulin production. Tests such as PGL-1 ELISA and immunochromatographic assays (e.g., ML Flow) can rapidly detect these antibodies and are useful for identifying high-risk populations and supporting clinical decision-making, especially in endemic regions^47^.

To improve diagnostic accuracy in PB forms, PGL-1 has been combined with recombinant proteins (e.g., LID-1) to enable simultaneous detection of multiple antibody classes. Nevertheless, antibody production in PB patients remains limited, and serological tests cannot replace clinical diagnosis. Other immunological markers, including inflammatory cytokines and interferon-gamma release assays, have been studied, but their applicability in routine practice remains under debate^48^.

Since serology may remain positive indefinitely after clinical cure, its role in post-treatment monitoring is limited. However, screening household contacts in highly endemic areas may help identify individuals at greater risk of developing the disease or harboring a higher bacillary load, warranting detailed clinical evaluation, regular follow-up, and clear education on symptoms and the role of complementary investigations^47^. Recent findings suggest that combining serological and molecular tests can enhance early detection in these contacts, supporting more targeted interventions^49^.

### Neurophysiological diagnosis

Leprosy is the leading cause of infectious neuropathy in many endemic regions, with peripheral nerve involvement accounting for most disease-associated disabilities^19,26^. Accordingly, neurophysiological evaluation is essential in detecting and monitoring nerve impairment.

Simplified neurological assessment using Semmes–Weinstein monofilament esthesiometry can be implemented in primary health care settings given its low cost, ease of application, and suitability fortrained health workers. This method has significantly contributed to the early detection of leprous neuropathy, identifying both initial disease manifestations and reactional episodes. Its routine use has expanded access to neurological diagnosis and been crucial in reducing disability rates^9^.

Electroneuromyography (ENMG) and nerve conduction studies provide valuable information by detecting changes consistent with segmental demyelination or axonal degeneration. Tuberculoid patients often exhibit mononeuropathy or multiple mononeuritis, while multibacillary forms evolve more frequently into diffuse polyneuropathy. These techniques also reveal subclinical abnormalities, enabling early therapeutic interventions to prevent irreversible nerve damage^50^.

Peripheral nerve ultrasonography complements these methods by identifying nerve thickening, intraneural edema, and changes in fascicular architecture^50^. Comparative studies indicate that this technique offers higher sensitivity and specificity than serological tests for early neural impairment, particularly in pure neural forms. It is especially valuable when cutaneous lesions are absent and peripheral nerves are the primary target. During reactional episodes, ultrasonography can confirm neuritis and monitor the progression of inflammation^51^.

Despite diagnostic advantages of ENMG and nerve ultrasonography, their availability remains limited in many endemic areas due to high costs and the need for specialized personnel^40^. Nevertheless, in reference centers, these tools are essential for early diagnosis, longitudinal follow-up, and therapeutic decision-making, contributing to the prevention of permanent deformities.

## Treatment and management

### Multidrug therapy (MDT)

The WHO recommends multidrug therapy (MDT), which combines rifampicin, dapsone, and clofazimine^33^. This regimen, also known as polychemotherapy (PQT), is distributed free of charge worldwide and remains the first-line treatment due to its high efficacy and contribution to reducing global disease prevalence over recent decades^33^.

In Brazil, since 2021, all leprosy cases have been managed with a unified MDT regimen (PQT-U), which includes rifampicin, dapsone, and clofazimine for both PB and MB patients. However, unlike the WHO recommendation of a uniform six-month regimen for all cases, Brazil maintains treatment duration according to clinical classification: PB patients receive six supervised doses within nine months, while MB patients undergo 12 supervised doses over a period of up to 18 months^31^.

Rifampicin is a bactericidal antibiotic that inhibits DNA-dependent RNA polymerase in *M. leprae*, thereby blocking RNA synthesis and inducing bacterial death. Dapsone acts as a bacteriostatic agent by inhibiting dihydropteroate synthase, disrupting folate synthesis, which is essential for bacterial replication and metabolism, thus contributing to load reduction^52^. Clofazimine binds to bacillary DNA, inhibiting replication and generating reactive oxygen species, which provides a mild bactericidal effect. It also modulates immune response by inhibiting pro-inflammatory cytokines, thereby reducing inflammation in leprosy^53^. With adequate adherence, MDT achieves cure rates above 90%; however, its use may be associated with adverse effects such as hepatotoxicity (rifampicin), hemolytic anemia (dapsone, particularly in individuals with G6PD deficiency), and skin hyperpigmentation (clofazimine)^54^.

Monitoring drug resistance is critical. Mutations in the rpoB gene (conferring rifampicin resistance) and folP1 gene (conferring dapsone resistance) are increasingly reported^55^. Laboratory surveillance and the implementation of alternative regimens are essential to manage resistant cases and prevent the spread of resistant strains^55^. MDT remains central in halting transmission by drastically reducing the bacillary load in treated individuals. Nonetheless, the continued detection of new cases each year underscores the need for complementary strategies such as active case finding and prophylaxis for household contacts^33^. Administration of single-dose rifampicin to contacts of MB patients has shown favorable outcomes in some settings but has not been adopted in Brazil due to concerns over uncertain efficacy and potential acceleration of drug resistance^11^.

### Alternative regimens

Resistance to one or more MDT components poses a growing challenge, particularly concerning rifampicin, which is the cornerstone of treatment. Although global resistance rates remain relatively low, certain regions have reported higher recurrence rates^56^. Genetic analyses indicate that mutations in folP1 (dapsone), rpoB (rifampicin), and gyrA (ofloxacin) genes account for most resistance cases, underscoring the importance of molecular screening in clinical management^56^.

When resistance is confirmed or suspected—based on persistent lesions and positive bacilloscopy—the implicated drug should be replaced. Alternatives to rifampicin or dapsone include clarithromycin, minocycline, and fluoroquinolones (ofloxacin or moxifloxacin), often used in combination regimens^31^. For instance, minocycline has demonstrated favorable outcomes when combined with clofazimine and ofloxacin. Clarithromycin, due to its excellent tissue penetration, can replace rifampicin or dapsone in specific contexts, although barriers related to cost and availability may limit its use^31^.

Reports of ofloxacin resistance in some areas restrict the use of fluoroquinolones. Mutations in gyrA appear to underlie this resistance; however, further research is needed to determine its prevalence and impact^56^. In such settings, clarithromycin and minocycline are preferred alternatives. Implementation of epidemiological surveillance and molecular diagnostic methods to detect mutations in folP1, rpoB, and gyrA is critical to guide timely therapeutic interventions and prevent the spread of resistant strains^55^.

Therapeutic failure may occur even in the absence of genotypically detectable resistance. In a case-control study of patients who completed MDT, the presence of foamy granulomas, neural or perineural lymphocytic infiltrates, a histological bacteriological index ≥3+, and anti-PGL-I ELISA index ≥3.95 at the end of treatment were significantly associated with treatment failure. Detection of *M. leprae* DNA by qPCR in skin biopsies also strongly correlated with persistent infection. The combination of these markers yielded predictive accuracy of up to 95% for treatment failure^57^.

Bedaquiline, originally developed for multidrug-resistant tuberculosis, has emerged as a promising option for leprosy treatment. A recent proof-of-concept study demonstrated that an eight-week course of bedaquiline monotherapy led to rapid clearance of *M. leprae* and clinical improvement in patients with multibacillary leprosy. The treatment exhibited bactericidal activity comparable or superior to rifampicin, with no serious adverse events reported during the study period^58^. Despite these encouraging results, considerations regarding cost, accessibility, and potential cardiac side effects (QT prolongation) require careful patient selection and monitoring when implementing bedaquiline-based regimens.

Beyond treatment of active disease, preventive strategies are essential for comprehensive leprosy control. BCG vaccination has been extensively studied as an immunoprophylactic approach, particularly in hyperendemic regions with high transmission risk. Evidence suggests that BCG provides partial protection, with even greater efficacy when two doses are administered to household contacts of multibacillary cases. Thus, systematic BCG vaccination constitutes an important component of leprosy control efforts^10^.

In contrast, other immunoprophylactic approaches are under investigation, including novel vaccines and immunological adjuvants to enhance protective responses against *M. leprae*. Several experimental vaccines, based on purified antigens or recombinant platforms, have yielded promising results in preclinical studies. Nonetheless, large-scale validation of their safety and efficacy is still required before widespread implementation^59^.

Single-dose rifampicin chemoprophylaxis provides a complementary strategy to reduce leprosy incidence among contacts. Although field studies have demonstrated its efficacy, implementation must follow well-defined protocols with careful antimicrobial resistance monitoring to ensure continued effectiveness and prevent the emergence of resistant strains^59^. Regional variations in resistance patterns and healthcare infrastructure require tailored approaches to both treatment and prevention strategies.

### Management of leprosy reactions

T1R with acute neuritis requires immediate medical attention to prevent irreversible nerve damage. Treatment involves corticosteroids such as prednisone or dexamethasone. Prednisone is typically initiated at 1 mg/kg/day, with gradual tapering of approximately 10 mg every 15 days. Once the dose reaches 20 mg/day, it should be reduced by 5 mg every 15 days. Upon reaching 5 mg/day, this dose is maintained for 15 days, followed by 5 mg on alternate days for another 15 days. Corticosteroid therapy should be maintained for a minimum average duration of six months, with regular monitoring of neural function and drug-related side effects. Prior to initiating corticosteroids, prophylaxis against disseminated strongyloidiasis is recommended, especially in endemic areas, using either albendazole 400 mg/day for three consecutive days or a single dose of ivermectin at 200 mcg/kg^31^.

T2R require a different treatment approach. Thalidomide is the primary treatment due to its potent immunomodulatory effects, particularly in controlling systemic inflammation. The initial dose ranges from 100 to 400 mg/day, adjusted according to clinical response, with gradual tapering to avoid relapse. However, due to its teratogenicity, thalidomide is contraindicated in women of childbearing age unless strict contraceptive measures are enforced. When thalidomide cannot be used, alternatives include pentoxifylline—an anti-inflammatory agent with a lower risk of severe adverse effects—and corticosteroids. The latter, although effective, carry a higher risk of reaction recurrence upon withdrawal. In severe cases, particularly those with significant neural involvement or systemic manifestations, combination therapy with corticosteroids and thalidomide may be necessary, or corticosteroids alone if thalidomide is not a viable option^31^.

In refractory T1R or T2R cases with persistent neural impairment, alternative therapeutic strategies have been investigated. Immunobiological agents such as tumor necrosis factor-alpha (TNF-α) inhibitors—including infliximab and adalimumab—and Janus kinase (JAK) inhibitors such as tofacitinib have emerged as potential options^25,60^.

TNF-α inhibitors, such as infliximab and adalimumab, block the activity of TNF-α, a pro-inflammatory cytokine essential to the pathogenesis of various inflammatory conditions. Although their efficacy is well established in diseases such as rheumatoid arthritis and Crohn’s disease, their use in leprosy remains experimental. Significant concerns include an increased risk of opportunistic infections, notably reactivation of latent tuberculosis and infections caused by non-tuberculous mycobacteria, due to TNF-α suppression^25^.

JAK inhibitors such as tofacitinib interfere with cytokine signaling pathways, modulating inflammatory responses. While promising, data on their efficacy and safety in leprosy are limited. Similar to TNF-α inhibitors, risks include severe infections and the potential interference with *Mycobacterium leprae* clearance^60^.

Given the limited clinical experience and potential risks, these therapies should be used cautiously, under strict criteria, with careful benefit-risk assessment and close patient monitoring to ensure both safety and therapeutic efficacy.

## CONCLUSION

Leprosy remains a significant public health challenge, particularly in socially vulnerable regions. Although multidrug therapy (MDT) demonstrates high efficacy and is freely available in many countries, barriers such as delayed diagnosis, drug resistance, and social stigma continue to hinder sustained reductions in transmission^6^. The diverse clinical spectrum—from paucibacillary to multibacillary forms—reflects the complex interplay between innate and adaptive immunity, shaped by genetic and environmental factors. This dynamic largely determines the host’s ability to contain or permit proliferation of *Mycobacterium leprae*.

Susceptibility to leprosy reactions is multifactorial, involving immune response, bacillary load, and treatment-related factors. Early identification and timely intervention with corticosteroids or immunomodulatory agents are critical to prevent disability. Conversely, genetic studies identifying mutations in rpoB, folP1, and gyrA genes have informed the development of alternative therapeutic regimens for patients with suspected resistance to rifampicin, dapsone, or quinolones, highlighting the importance of molecular tools in epidemiological surveillance.

Despite therapeutic advances, persistently high incidence rates underscore the need to expand preventive strategies. BCG vaccination, especially among household contacts of multibacillary cases, has been associated with reduced risk of disease. Single-dose rifampicin chemoprophylaxis remains under discussion, and its widespread implementation should be based on robust efficacy and safety data, particularly concerning the potential emergence of resistant *M. leprae* strains.

Neurological rehabilitation, conducted by multidisciplinary teams, are key in reducing disability, enhancing autonomy, and promoting social reintegration, thereby mitigating disease-related stigma. Effective leprosy control requires integrated strategies that promote early diagnosis, expanded preventive measures, and interventions addressing social inequalities that perpetuate transmission.

## Data Availability

The anonymized dataset generated during this study is available from the corresponding author upon reasonable request.
